# A Reminder of Gender Diversity Within Scientific Literature: A Qualitative Perspective

**DOI:** 10.1007/s10508-025-03313-0

**Published:** 2025-09-18

**Authors:** Serena G. Pehlivanidis

**Affiliations:** Australian Research Centre in Sex, Health and Society, La Trobe University, NR6, Melbourne, 3806 Australia

## Introduction

Discourse surrounding gender diversity has become increasingly prominent globally, within both social and political spheres. Naturally, the academic space has followed suit, with a burgeoning reminder to reflect on the documented realities of those who actually live trans and gender diverse lives outside of the binary. Qualitative documentations of the trans and gender diverse communities within scientific literature are powerful in that they demonstrate the expansive, complex, and very real existence of gender diversity. It is with this notion at the fore that the prompt “How many genders are there?” will be discussed in this commentary. In doing so, it will be asserted that gender is not a finite binary system to be enforced, but rather, a dynamic and personal identity category that should be explored with curiosity. Further, a case will be made for the importance of continued qualitative explorations in this space, to ensure the dignity and well-being of trans and gender diverse individuals worldwide.

## Gender in Science

Gender terminology in science has long been at the center of debates regarding its separation and conflation with sex, leading to current understandings that offer complex and nuanced views of the gender/sex relationship (Hyde et al., 2019; van Anders & Dunn, 2009; van Anders et al., 2015). For the purpose of this commentary, sex refers to biological characteristics, such as X and Y chromosomes, hormonal differences, and pre- and post-natal sexual differentiation that collectively determine sex assigned (and, ultimately, presumed) at birth. Although years of scientific research have shown the many variations in the above-mentioned biological characteristics, Western medical approaches to sex use dichotomous categorizations, namely "male" or "female" (Lee et al., 2016).

Gender identity, recognizing that gender is a socially defined and performed construct within society, denotes an individual’s sense of self and experience of gender. Gender is best understood as a dynamic, socially constructed, and contextually situated identity category, shaped by psychological processes, cultural norms, interpersonal interactions, and other intersecting factors (Cole, 2009; Hyde et al., 2019). Over time, psychological understandings of gender have shifted from a binary and essentialist understanding, to one that recognizes gender as plural, fluid, and informed by lived experience. Oftentimes, one’s experience of gender is in alignment with their sex characteristics (as defined by social norms and expectations of gender/sex experiences) and thus one is cisgender. For example, a cisgender woman would have female sex characteristics that align with a traditionally binary "woman" gender experience. Those who do not experience this alignment in sex and gender identity are broadly referred to as being transgender and gender diverse (hereafter referred to as trans; Connolly et al., 2016). A trans man may have a gender experience traditionally described as being that of a "man"; however, may possess "female" sex characteristics. Further, there are gender identities that are non-binary, which is when an individual does not identify in ways that align with the dichotomous categories of man and woman (Tate et al., 2014). Non-binary experiences similarly sit under the expansive umbrella of transgender experiences (Connolly et al., 2016). The above understandings of gender are those that have been studied and developed over time within scientific literature and provide the basic foundations for modern scientific explorations of the human social and psychological experience.

## Gender Diversity in Society

Conceptualizations of gender variance beyond the binary have a well-documented history and have existed long before the establishment of rigorous scientific research methodology. Gender diversity contrary to the dichotomous "man"/"woman" gender narratives often upheld in Western society, exist within many cultures worldwide; for instance, the documented and oral histories of Sistergirls and Brotherboys in Indigenous Australian communities (Riggs & Toone, 2017). Other such cross-cultural, historical documentations of gender diversity beyond binary categorizations include Two-Spirit identities in Native American cultures, Hijra of South Asia, Fa’afafine of Samoa, and Kathoey of Thailand, to name a few (Nanda, 2014; Towle & Morgan, 2002).

Lived experiences of those who do not "fit" this binary are similarly seen within modern Western society and quantitative insights confirm the presence and prevalence of gender diversity today. In younger Australian LGBTQ+ populations, we see gender diversity at rates of 27.2% (Hill et al., 2021). Specifically, 19.5% of Australian LGBTQ+ youth identify as non-binary, 6.5% identify as trans men, and 1.2% identify as trans women. In broader Australian LGBTQ+ populations, 21.8% are gender diverse (Hill et al., 2020). Though the Australian census has not historically collected data on gender diversity in the country, the Household, Income and Labor Dynamics in Australia survey’s most recent data collection shows a notable number of Australians identify with gender categories other than man or woman, with 0.7% to 1.5% of this large longitudinal sample identifying as trans (Saxby et al., 2025). Recent estimates released by the Australian Bureau of Statistics discuss similar figures, in which 0.9% of the population aged 16 years and over identify as trans (Australian Bureau of Statistics, 2022).

## The Reality of Gender Expansiveness in Qualitative Literature

Within the social sciences, qualitative researchers play a vital role in investigating underlying societal patterns and lived experiences of people in society. Qualitative inquiry is often guided by real-world observations and insights that must be recorded and assessed in an effort to describe social patterns and behaviors (Babbie, 2008). Such methods are commonly used to develop and enhance nuanced understandings of individual and group behaviors, relationships, and broader psychological processes. A space in which qualitative research has and continues to be particularly useful is the gender identity space. The reason qualitative inquiry is especially helpful in this instance is that this method allows for in-depth and nuanced interpretations of complex experiences (Babbie, 2008). Suen et al.’s (2020) qualitative study of the limitations of sexual orientation and gender identity survey questions is a prime demonstration of such an approach in this population. The study’s findings confirm the complexity of trans identities and ratify the need for fluid conceptualizations of gender identity in research and clinical settings. Even within cisgender cohorts, individuals hold their own unique relationships with gender (Freshley et al., 2024). However, this fluidity in lived experience is especially true for trans populations, given that society’s guiding binary principles, which often dictate gender norms and expectations, hold little space for trans lives (Beischel et al., 2024). So oftentimes, trans individuals must pave the way for themselves.

Trans experiences are expansive, that is to say, they cannot be painted with a broad brush. Qualitative studies have been especially crucial in understanding the lived experiences of gender diversity within society and solidifying the idea that gender diversity captures a spectrum of experiences (Conlin et al., 2019; Fiani & Han, 2019). This idea subverts the traditional, Western, medicalized view of trans lives, in which all trans individuals seek to medically transition to the "opposite” binary gender (Matsuno & Budge, 2017). This dated perspective assumes the narrative that all trans people are "born in the wrong body" and simultaneously pathologizes trans identities. Indeed, this conceptualization of transness resonates with some individuals; however, emerging qualitative literature has found that these experiences are not necessarily shared by all trans individuals. Rather, there are significant portions of trans communities who do not ascribe to the gender binary and whose experiences offer insight into the diversity of peoples' relationships with gender and their bodies (e.g., Burstall et al., 2024; Conlin et al., 2019). Despite there being similarities between trans binary and trans non-binary narratives, there are notable differences in identity formation, as well as personal, interpersonal, and systemic challenges faced by non-binary individuals (Fiani & Han, 2019). The largest Australian survey of LGBTQ+ individuals to-date (n = 6,782) reports that 13.6% identify as non-binary, compared to 4.4% trans men and 4.2% trans women (Hill et al., 2020).

This notable part of the trans population describe experiences that demonstrate the personal nature of gender diversity and there being no singular narrative to being trans. Evidence indicates that some gender diverse experiences are not homogenous across all trans individuals, such as the masculinization of one’s chest through “chest binding” to create a masculine body contour. This practice, though a pivotal step for some in their gender journey, is not necessarily a desired or achievable goal for all transmasculine individuals (Pehlivanidis & Anderson, 2024a, 2024b). Similarly, and more broadly, the way non-binary individuals experience their bodies and describe their physical ideals are extremely diverse (Burstall et al., 2024). Such heterogeneity of bodily experiences are common across the binary and non-binary spectrum (Fiani & Han, 2019). For instance, some trans individuals value and attempt to physically "pass"; a controversial notion that trans people must reach a cis-normative gender presentation as opposed to being visibly trans. Other trans individuals, both trans binary and non-binary, hold the view that the pursuit of "passing" only further ingrains a dichotomous gender system and do not desire to change their physical appearance to fit cis-normative gender expectations (Anderson et al., 2025).

Intersectional experiences, particularly cultural norms and contexts, contribute additional nuance to the lived realities of gender diversity. Qualitative reports are particularly helpful in creating understandings of the complex group differences that exist within trans communities. For instance, an emerging body of qualitative literature suggests that the gender diversity formation and experiences of trans individuals who live with a disability differ from those of their trans counterparts who do not (e.g., Baril et al., 2020). Similar instances of difference are seen in qualitative descriptions of gender diversity realities of trans neurodivergent populations (Peachey & Crane, 2024). Further, growing qualitative accounts of trans people of color’s experiences with gender identity, transition, and affirmation are being seen in Australian literature (e.g., Ussher et al., 2022). Such works are similarly emerging beyond Australian contexts, for instance, de Vries and Sojka’s (2022) paper that used interviews to understand how racial identities shift in tandem with shifts in gender identity and ultimately tied racialized gender to the power structures that exist at the intersections of gender, race, sexuality, and nationality. Academic understandings of these intersections with gender diversity are still emerging and continually developing. However, it is evident that gender diversity is an ever fluid and evolving reality that is shaped by a number of life factors and individual lived experience. Hence, it cannot be understated how critical qualitative efforts are in continuing to form comprehensive understandings of and providing the foundations for gender identity frameworks that describe all lived realities.

## The Importance of Continued Exploration

As academic conceptualizations of gender diversity continue to develop and societal discourse around gender diversity becomes more prominent, it is clear that our understandings of gender are incomplete. Current literature evidences the existence of more than two dichotomous genders; however, the need for continued exploration is evident. Qualitative research demonstrates the ever expansive, overlapping, and diverging experiences of those in trans communities. Continuing investigations of group variation, such as the intersectional influences of cross-cultural and neurodiverse experiences on gender development, are important in furthering the existent literature. Such advances have notable implications for societal outcomes and well-being.

As the research currently stands, trans populations navigate particular stressors pertinent to their social and psychological well-being (Hendricks & Testa, 2012; Matsuno et al., 2024; Testa et al., 2015). Trans populations face heightened levels of discrimination and stigma across many life facets, for instance, in seeking general healthcare and in accessing gender-affirming care (Strauss et al., 2017; Zwickl et al., 2024). The threat of gender diversity-based violence is similarly pertinent, with Australian trans youth reporting forms of violence across familial, educational, and public domains (Hill et al., 2021). These stressors contribute to the mental health outcomes of this community, where depression rates sit at an average of 46.2% in trans Australian youth (Hill et al., 2021).

Group differences in these outcomes confirm the heterogeneity of experience within the community, for example, very high psychological distress is seen at rates of three quarters (75.8%) in trans men, 74.9% in non-binary participants, and at a slightly lower, but still unacceptably high rate of 65.6% in trans women (Hill et al., 2020). Further, those at the intersections of gender diversity and other factors face quite detrimental outcomes. For instance, trans Australians with a disability (Amos et al., 2023) or who are culturally or linguistically minoritized (Tan et al., 2025) are particularly vulnerable to instances of abuse and social exclusion.

In an effort to sufficiently address such outcomes, and in response to the recent global, social, and political discourse calling for finite limits on gender, we sustain that the question, “How many genders are there?” cannot be answered with a simple numerical value. Qualitative insights imply that the more pertinent question may be: “How can psychological science represent and affirm the reality of gender diversity in our communities?” Future research must continue to center lived experience, particularly among historically marginalized communities, and adopt intersectional frameworks that recognize how gender interacts with culture, class, disability, and sexuality. The role of psychology in this context is not only descriptive, but also normative; we are called to challenge outdated models and support inclusive practices across science, healthcare, education, and social policy. Most critically, a nuanced, evidence-based understanding of gender identity is essential for the well-being and dignity of all trans individuals worldwide.
